# Influence of methylation and demethylation on plant uptake of emerging contaminants

**DOI:** 10.1016/j.envint.2022.107612

**Published:** 2022-11-01

**Authors:** Yaxin Xiong, Qingyang Shi, Nathan D. Sy, Nicole M. Dennis, Daniel Schlenk, Jay Gan

**Affiliations:** Department of Environmental Sciences, University of California, Riverside, CA 92521, USA

**Keywords:** CECs, Plant accumulation, Transformation products, Methylated/demethylated derivatives

## Abstract

Contaminants of emerging concern (CECs) as well as their transformation products (TPs) are often found in treated wastewater and biosolids, raising concerns about their environmental risks. Small changes in chemical structure, such as the addition or loss of a methyl group, as the result of methylation or demethylation reaction, may significantly alter a chemical’s physicochemical properties. In this study, we evaluated the difference in accumulation and translocation between four CECs and their respective methylated or demethylated derivatives in plant models. Suspended *Arabidopsis thaliana* cell culture and wheat seedlings were cultivated in nutrient solutions containing individual compounds at 1 mg/L. The methylated counterparts were generally more hydrophobic and showed comparative or greater accumulation in both plant models. For example, after 1 h incubation, methylparaben was found in *A. thaliana* cells at levels two orders of magnitude greater than demethylated methylparaben. In contrast, the demethylated counterparts, especially those with the addition of a hydroxyl group after demethylation, showed decreased plant uptake and limited translocation. For example, acetaminophen and demethylated naproxen were not detected in the shoots of wheat seedlings after hydroponic exposure. Results from this study suggest that common transformations such as methylation and demethylation may affect the environmental fate of CECs, and should be considered to obtain a more comprehensive understanding of risks of CECs in the environment.

## 1. Introduction

Contaminants of emerging concern (CECs) refer to contaminants that are recently discovered in the environment and may pose potential adverse effects, such as developmental toxicity and endocrine disruption, to non-target organisms and human health at environment-relevant concentrations (Kidd et al., 2007; Lovingood et al., 2018; Oaks et al., 2004). Because of their widespread use, CECs are ubiquitously present at trace levels in treated wastewater and biosolids. (Ashfaq et al., 2018; Česen et al., 2019; Evgenidou et al., 2015; Lovingood et al., 2018; Poustie et al., 2020; Zhi et al., 2020). Many CECs contain reactive functional groups such as hydroxyl, carboxyl, and amide, making them susceptible to biotic and abiotic transformations during treatment at wastewater treatment plants (WWTPs) (Bulloch et al., 2015; Evgenidou et al., 2015; Ren et al., 2021). Therefore, in addition to the parent form of CECs, transformation products (TPs) are also often present in treated wastewater and biosolids, sometimes at even higher concentrations (Rüdel et al., 2013). Treated wastewater and biosolids have been increasingly applied to agricultural lands in recent years in beneficial reuse practices, which serves as a conduit for plants to be contaminated with CECs and their TPs, posing potential human health and ecological risks (Fu et al., 2019; Li et al., 2022; Shahriar et al., 2021; Sharma et al., 2020).

**Fig. 1.**
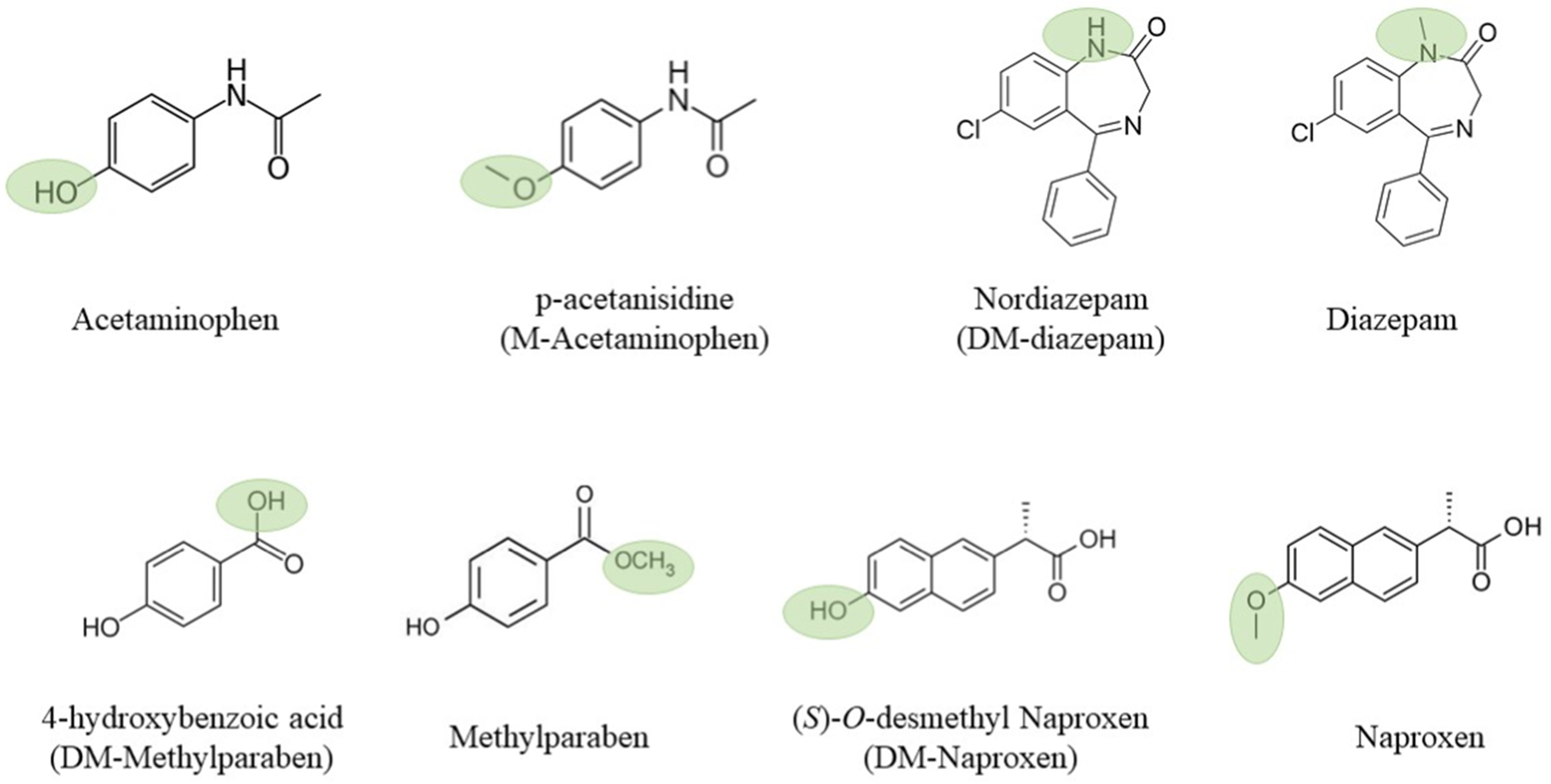
Chemical structures of the target compounds considered in this study; methylated or demethylated part indicated with a green circle.

Methylation and demethylation are among the most common transformation reactions for many CECs. Biotic demethylation is a phase I metabolism process facilitated mainly by cytochrome P450 enzymes that are ubiquitous in organisms (Chuang et al., 2018; Hagel, 2010; Miners et al., 1996; Yu and Yang, 2010). For example, as a common pharmaceutical itself, nordiazepam (demethylated diazepam or DM-diazepam) is also a demethylated metabolite of diazepam excreted after oral administration in humans (Onof et al., 1996) (Fig. 1). Likewise, demethylation can convert naproxen to *O*-desmethyl naproxen (DM-naproxen), and methylparaben to 4-hydroxybenzoic acid (DM-methylparaben) through microbially mediated phase I metabolism (Wolfson et al., 2019, 2018) (Fig. 1). Abiotic demethylation of herbicides and some CECs was also observed after advanced oxidation processes during wastewater treatment (Konstantinou et al., 2014). Therefore, demethylated counterparts are among the most commonly observed TPs of CECs. Biotic methylation is a phase II metabolism mediated by methyltransferases (Bártíková et al., 2015). Methylated acetaminophen, i.e., p-acetanisidide (M-acetaminophen) (Fig. 1), is a major metabolite of acetaminophen in soil (Li et al., 2014). Methyl triclosan is the primary TP of the antimicrobial triclosan after WWTP treatment (Bozlee, 2017). Tetrabromobisphenol A (TBBPA), a brominated flame retardant, was found to be *O*-methylated by microbes, as well as in pumpkin plants and earthworms (Chen et al., 2017; George and Häggblom, 2008; Hou et al., 2018). Naturally occurring methyl iodide can also cause the abiotic methylation of phenolic contaminants (Hou et al., 2019a).

The addition or loss of a methyl group during transformations alters a compound’s physicochemical properties (Wang and Kelly, 2017), which may subsequently affect its fate and risk in the environment. As uptake of CECs into plants is known to depend closely on a chemical’s physicochemical properties, such as lipophilicity (i.e., *K*_ow_) (Li et al., 2019; Pérez et al., 2022; Wu et al., 2013), it may be hypothesized that methylation or demethylation changes a chemical’s behavior and fate in the soil–plant continuum. Despite their frequent occurrence, the environmental significance of simple transformation reactions such as methylation and demethylation is often overlooked. In this study, we compared plant accumulation and translocation of four pairs of compounds differing only in a methyl group in their structures using two plant models, *Arabidopsis thaliana* cells and wheat seedlings. Four CECs (acetaminophen, diazepam, methylparaben, and naproxen) and their respective methylated or demethylated counterparts (M-acetaminophen, DM-diazepam, DM-methylparaben, and DM-naproxen) were chosen as the test compounds because of their widespread use and occurrence in the environment (Calcaterra and Barrow, 2014; He et al., 2017; Nowak et al., 2021). Of these compounds, DM-diazepam is not only a TP of diazepam, but also a pharmaceutical itself, and DM-methylparaben is not just a TP of methylparaben, but also an industrial raw material (Nowak et al., 2021; Sacre et al., 2017). Results from this study contribute to a better understanding of the implications of simple transformation reactions such as methylation and demethylation on the environmental behavior and potential risks of CECs.

## 2. Materials and methods

### 2.1. Analytes, surrogates, and solvents

All analytical standards were purchased with reported purities ≥ 98 %. Acetaminophen, diazepam, DM-diazepam, *d*_5_-diazepam (used as surrogate for diazepam and DM-diazepam), naproxen, DM-methylparaben and methylparaben were purchased from Sigma-Aldrich (St. Louis, MO). M-Acetaminophen was purchased from Santa Cruz Biotechnology (Dallas, TX). DM-naproxen and *d*_4_-methylparaben (used as surrogate for DM-methylparaben and methylparaben) were purchased from Toronto Research Chemicals (Toronto, Ontario, Canada). *d*_4_-Acetaminophen (used as surrogate for acetaminophen and M-acetaminophen) and *d*_3_-naproxen (used as surrogate for naproxen and DM-naproxen) were purchased from C/D/N Isotopes (Pointe-Claire, Quebec, Canada). HPLC-grade methanol, acetonitrile, methyl *tert*-butyl ether (MTBE) and acetone were purchased from Fisher Scientific (Fair Lawn, NJ). Ultrapure water was generated by an in-house Milli-Q water purification system (Millipore, Carrigtwohill, Cork, Ireland). Radioisotope labeled compounds were not used in this study, and therefore the uptake efficiency or mass balance of the target compounds in plants could not be derived.

### 2.2. Uptake in Arabidopsis thaliana cells

The *A. thaliana* cell suspension (cell line T87, CCL84839) was obtained from the Arabidopsis Biological Resource Center at Ohio State University (Columbus, OH) and was maintained in the laboratory at 24 °C and 130 rpm in NT-1 media with constant light (Text S1). An aliquot (5 mL) of the *A. thaliana* cell culture was added to fresh, auto-claved NT-1 media (25 mL), and incubated for 3 d, after which each cell suspension was spiked with individual compounds to arrive at an initial concentration of 1 mg/L. Control treatments included positive and negative control groups containing CECs spiked in nonviable cells (autoclaved at 121 °C for 45 min), CECs in blank culture solution, or viable cell culture solution without CECs. Each treatment was prepared in triplicate, and was sampled at 1, 3, 6, 11, 24, 48, and 96 h. Entire samples were transferred to polypropylene centrifuge tubes (50 mL) and were immediately centrifugated at 3500 rpm for 30 min. The cell matter was stored at −80 °C until further analysis, and the supernatant was transferred into a 40 mL glass bottle and stored at −20 °C until further analysis.

### 2.3. Uptake in wheat seedlings

Wheat seedlings used in this study were germinated from seeds to avoid potential background contamination. Sterilized seeds were germinated on a moist filter paper on a tray in the dark at room temperature. The tray was then transferred into a growth chamber (24 °C, 16:8h light:dark ratio) after 2 d for seedlings to grow. When the seedlings grew to about 5 cm in height, they were transplanted into a 50-mL polypropylene centrifuge tube wrapped in aluminum foil (to prevent light exposure to the roots) and then cultivated in the growth chamber. Initially filled with water, the solution in the tubes was replaced to 1/4 strength and then 1/2 strength Hoagland^®^ nutrient solution at 2-day intervals to allow wheat seedlings gradually acclimating to the nutrient media. Once acclimated, the media were replaced with 30 mL fresh 1/2 strength Hoagland^®^ nutrient solution spiked with a single compound of interest from individual stocks (1000 mg/L) to reach a nominal chemical concentration of 1 mg/L. Water was added to each tube every other day to make up the lost water throughout the incubation experiment.

Triplicate containers were sacrificed at 0, 3, 6, 12, 24, 48, 96, 168 and 240 h after the treatment. Plants were rinsed with deionized water, dried with paper towels, and separated into roots and shoots. The nutrient solutions remained in the centrifuge tubes and separated plant tissues were stored at −80 °C until further analysis. Transpiration stream concentration factors of target compounds were not measured in wheat seedlings in this study, due to challenges in collecting adequate amount of xylem sap for analysis.

### 2.4. Sample preparation

Deuterated compounds were used as surrogates during extraction for QA/QC. Extraction of nutrient solutions from *A. thaliana* cells and wheat seedlings was carried out using a similar method to a previous study (Wu et al., 2012), with minor modifications. Briefly, 50 μL of the surrogate solution (10 mg/L) was added to a 5 mL aliquot of nutrient solution. The nutrient solution samples were then extracted by HLB cartridges (6 mL, 150 mg). Methanol, and then water, 7 mL each, were added to each HLB cartridge for precondition, followed by the addition of the sample and then 5 mL of 5 % methanol in water for clean-up. For elution and collection of the target analytes, a final pass-through of 15 mL methanol was performed. The resulting methanol eluent was collected in a glass tube, dried using a nitrogen evaporator, reconstituted in 1 mL methanol–water mixture (1:1, v/v), and filtered through a 2 mm PTFE filter into a 1.5 mL HPLC vial for instrumental analysis.

Plant cell matter and wheat tissues were freeze dried at −50 °C for at least 72 h to remove moisture and weighed. Before extraction, 50 μL of the corresponding deuterated compound (10 mg/L) was added to each sample as the recovery surrogate. Samples were firstly extracted with 10 mL MTBE via sonication for 30 min. The sonication process was then repeated with 10 mL fresh MTBE one additional time and 10 mL acetonitrile twice. Extracts from the extraction were combined and dried by a nitrogen evaporator, followed by reconstitution in 1 mL methanol and dilution with 20 mL water. The resulting liquids were cleaned up with HLB cartridges using a similar protocol as described above. The final extracts were dried under a gentle nitrogen gas flow, reconstituted in 1 mL methanol: water (1:1, v/v), and filtered through a 2 mm PTFE filter before instrument analysis.

### 2.5. UPLC-QqQ-MS/MS analysis

Analytical methods for all compounds were established on a Waters ACQUITY ultra-performance liquid chromatography (UPLC) with a Waters Triple Quadrupole mass spectrometer (QqQ-MS/MS) (Waters, Milford, MA). An ACQUITY BEH C18 column (100 × 2.1 mm i.d., 1.7 μm; Waters, Milford, MA) in a 40 °C column compartment was used for chromatographic separation. The mobile phases (A and B) were 0.01 % formic acid in water and methanol, respectively. Extracts were injected (5 μL) and separated along the following solvent gradient: 0–1 min, 5 % to 40 % B; 1–2 min, 40 % to 90 % B; 2–4 min, 90 % to 95 % B; and 4–6 min, re-equilibration with 5 % B. The flow rate was 0.3 mL/min. The MRM transitions of all target compounds were optimized and are provided in Table S1. Data were processed by TargetLynx XS software (Waters, Milford, MA).

### 2.6. Quality assurance and quality control

The recoveries and limits of quantification (LOD) for the target compounds are provided in Table S2. Method blanks and matrix blanks were included to check for possible contamination and carryover. One solvent blank and one check standard (100 μg/mL) were injected after every 10 samples. No target analytes were detected in the solvent blanks. Data were calculated as mean ± standard deviation (SD). Systematic differences were evaluated at a significance level of 0.05 and the data were processed and graphed using SPSS Statistics 27 (IBM Corp, Armonk, NY) and Prism 9 (GraphPad, La Jolla, CA), respectively.

## 3. Results and discussion

### 3.1. Accumulation in A. thaliana cells

No significant difference was found in the biomass between the treated groups and control groups for experiments using *A. thaliana* cells. No target analytes were detected in the method blanks. The concentrations of target analytes in control groups with no cells, or with nonviable cells, varied in the range of 93.4–116.0 % of the spiked concentration at the end of exposure as compared to the initial concentrations, suggesting stability of these compounds under abiotic conditions.

**Fig. 2.**
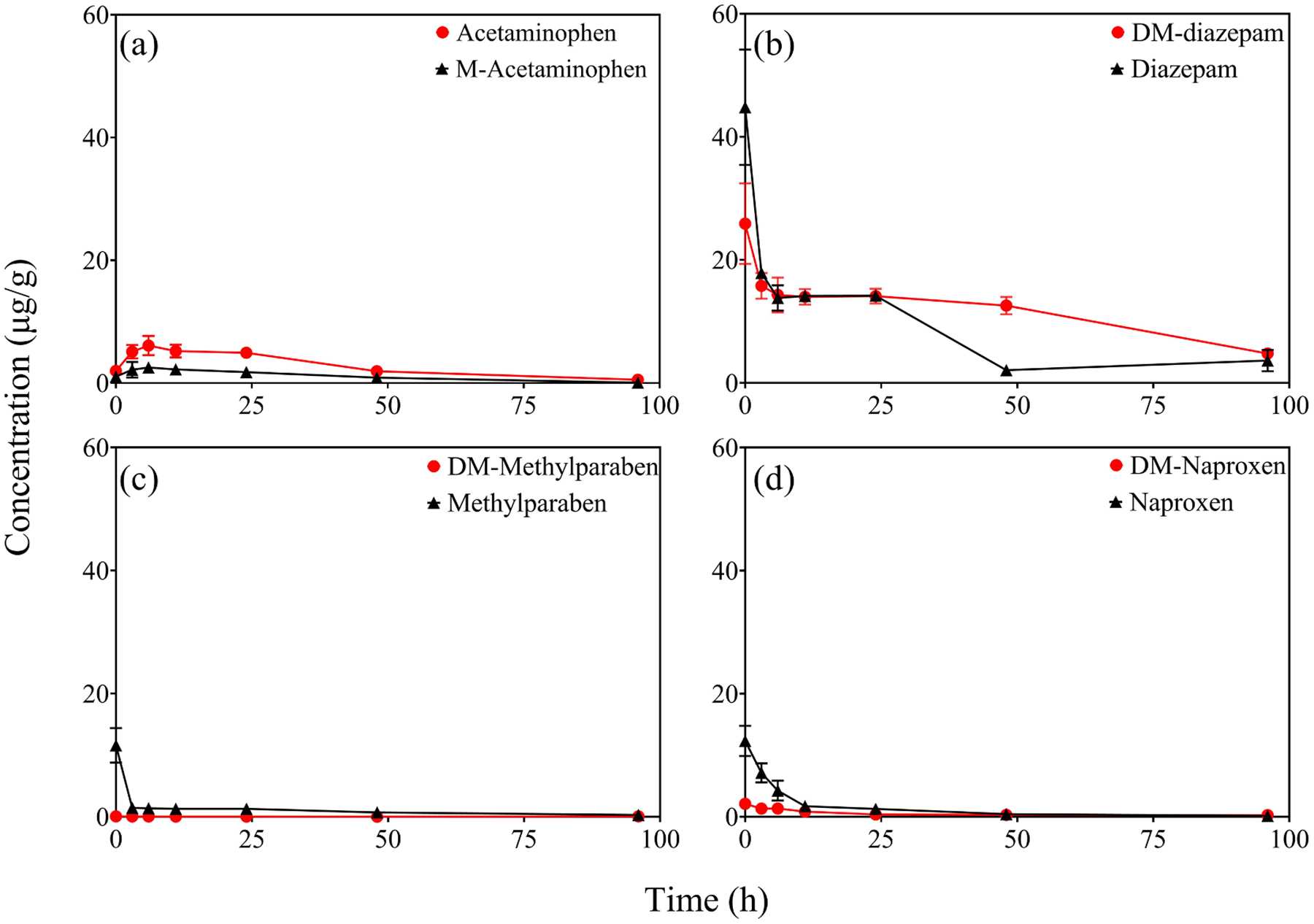
Accumulation of CECs and their methylated or demethylated transformation products in *A. thaliana* cells.

The individual compounds were found to be taken up by live *A. thaliana* cells. The levels in the plant cell matter reached maxima within 3 h for all compounds except acetaminophen, which exhibited the highest accumulation at 6 h into the incubation (Fig. 2). The level of chemicals in the cell matter decreased quickly thereafter. At the end of 96-h cultivation, the level in the cell matter was < 0.3 μg/g (dry weight, d.w.) for most compounds, suggesting rapid metabolism in viable plant cells and likely excretion into the aqueous medium. Among the different compounds, DM-diazepam and diazepam appeared to be accumulated to higher levels than the other compounds and were also more recalcitrant to metabolism. After 96 h of incubation, 4.78 ± 0.90 μg/g of DM-diazepam or 3.63 ± 1.74 μg/g of diazepam still remained in the *A. thaliana* cells. In previous studies, CECs including acetaminophen, diazepam and naproxen were found to be readily metabolized in different plant species (Dudley et al., 2019; Fu et al., 2017b; Sun et al., 2019).

The CECs and their methylated/demethylated derivatives showed different accumulation potentials in *A. thaliana* cells. For example, acetaminophen was detected in the *A. thaliana* cells at significantly higher concentrations than M-acetaminophen at any given sampling time point (*p* < 0.05). After 6 h of incubation, 6.10 ± 1.57 μg/g of acetaminophen was found in the *A. thaliana* cells, while the level was only 2.52 ± 0.57 μg/g for M-acetaminophen (Fig. 2a). In comparison, methylparaben was found to accumulate more than DM-methylparaben at all sampling time points. For example, at 1 h, methylparaben was found at 11.6 ± 2.81 μg/g in the cell matter, while DM-methylparaben at only 0.06 ± 0.00 μg/g. The difference in accumulation by *A. thaliana* cells between DM-methylparaben and methylparaben may be partly attributed to the fact that DM-methylparaben was present mostly in an ionized form in the nutrient media (Table 1). Negatively charged chemicals are known to not easily cross the negatively charged cell walls and membranes and are limited in their plant uptake (Manasfi et al., 2021; Shi et al., 2022). Like methylparaben, higher concentrations of naproxen than DM-naproxen were also detected in the cell matter throughout the exposure time (Fig. 2d). After 1 h of incubation, 12.31 ± 2.46 μg/g of naproxen was found to be in the cell matter, while the level was only 2.10 ± 0.40 μg/g for DM-naproxen. However, no statistically significant difference was observed between diazepam and DM-diazepam in their levels in *A. thaliana* cells during the exposure experiment. This may be attributed to the fact that log *K*_ow_ of DM-diazepam is similar to that of diazepam (Table 1).

As the test chemicals were taken up by *A. thaliana* cells, the levels of CECs and their methylated or demethylated derivatives in the culture media concurrently decreased. In the culture media, the concentration of DM-methylparaben and methylparaben, and DM-naproxen and naproxen all decreased rapidly, and their level fell below the detection limit after just a few hours into the incubation (Fig. S1). In comparison, the decrease of acetaminophen and M-acetaminophen, and DM-diazepam and diazepam was relatively slower, with 0.12–0.40 mg/L, or 12–40 % still remaining in the cell culture media after 48 h of exposure. The dissipation of CECs and their methylated or demethylated derivatives in the culture media was further fitted to the first-order decay model, and the fit was generally good, with R^2^ > 0.63. The half-life *T*_1/2_ was then calculated from the first-order rate constant (Table 2). The estimated *T*_1/2_ values were very small for methylparaben, DM-methylparaben, and naproxen. The dissipation of DM-naproxen was so rapid that *T*_1/2_ could not be derived. Methylation appeared to increase *T*_1/2_ for acetaminophen and DM-diazepam, with statistically significant difference (*p* < 0.05 between acetaminophen and M-acetaminophen, and *p* < 0.001 between DM-diazepam and diazepam).

A mass balance approach was not followed in this study, as subsequent transformation products in the *A. thaliana* cells were not characterized. Given that the compounds considered in this study were stable under abiotic conditions, the rapid dissipation in the culture media and limited accumulation in the *A. thaliana* cells suggested that the CECs and their methylated or demethylated counterparts underwent rapid metabolism in the *A. thaliana* cells. In the case of acetaminophen, methylparaben, and naproxen, demethylation introduced a hydroxyl or carboxyl group into the molecule. As shown in previous studies, compounds with a hydroxyl or carboxyl functional group can undergo rapid conjugation with various biomolecules in plants (Fu et al., 2017b; Hou et al., 2019b; Huber et al., 2012). The conjugated intermediates are substantially larger in molecular size and may become “immobilized” once formed in the *A. thaliana* cells (Burken, 2004; Grzam et al., 2006; Huynh et al., 2018). Future research should consider the formation of conjugates for demethylated compounds and understand the fate and risks of such plant-origin conjugates.

### 3.2. Accumulation and translocation in wheat seedlings

Uptake and translocation of the paired compounds were further measured in wheat plants grown hydroponically in nutrient solutions. Roots and shoots of wheat seedlings were collected and analyzed separately to understand the in-plant translocation. Target CECs and their methylated or demethylated counterparts showed great stability in hydroponic solution without wheat seedlings, with recoveries ranging from 99.1 to 125.7 % of the initial spiked concentration after 240 h incubation. No compounds of interest were detected in the untreated hydroponic solution or wheat seedlings.

**Fig. 3.**
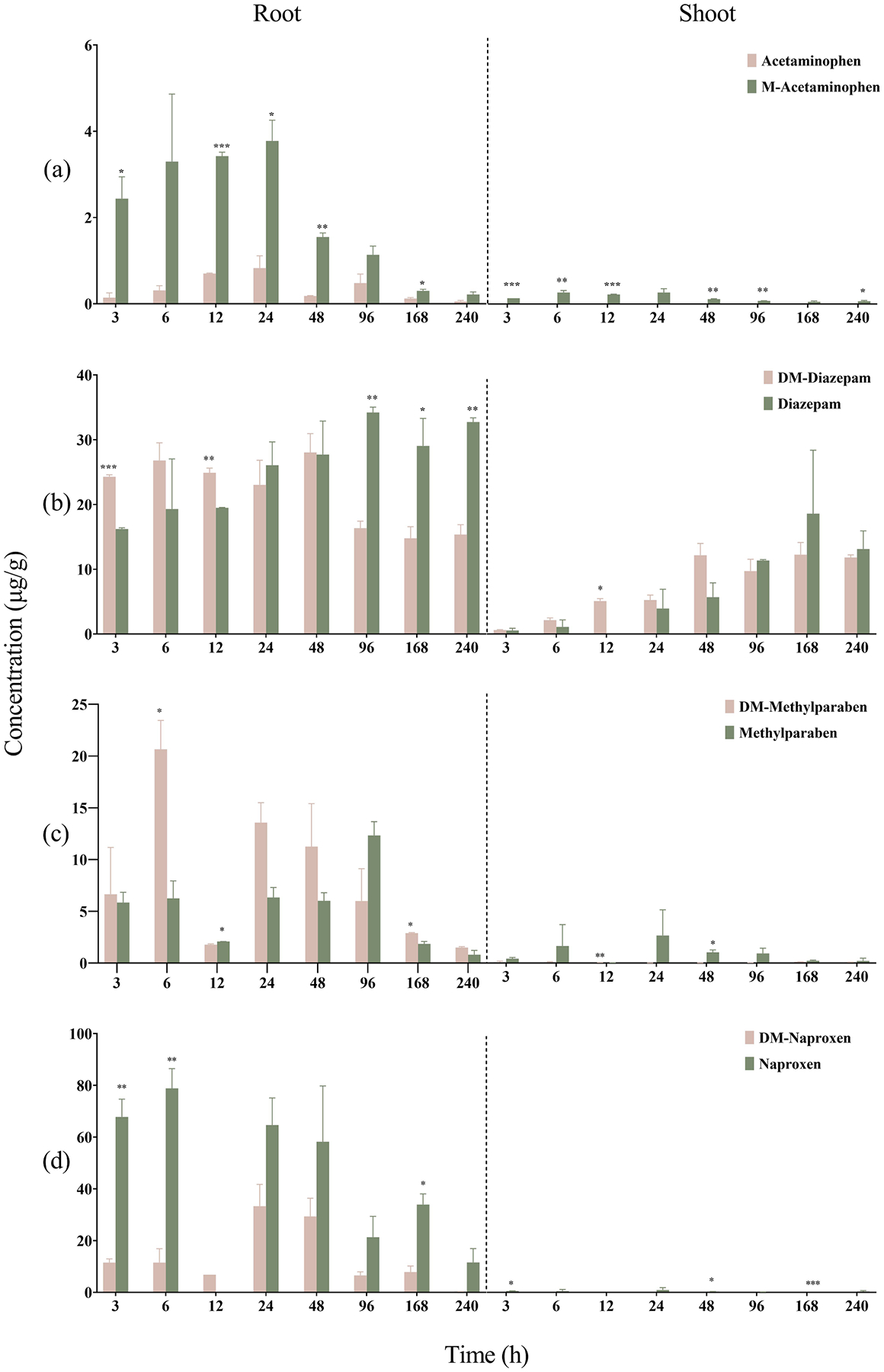
Accumulation of CECs and their methylated or demethylated counterparts in wheat roots and shoots.

In general, the level of chemicals in the plant tissues first increased and then decreased, suggesting uptake into the roots from the hydroponic media, followed by translocation from roots into shoots and/or metabolism in the plant. All CECs and their methylated or demethylated TPs were detected in wheat roots, and the concentrations were much higher than those in shoots, indicating generally limited translocation (Fig. 3). Among the different CECs, acetaminophen and DM-naproxen were not detected in wheat shoots, while DM-methylparaben was only found occasionally at trace levels. The accumulation of acetaminophen was also limited in the roots, which may explain its absence in the shoots. From a previous study (Fu et al., 2017b), after formation from naproxen through demethylation, DM-naproxen was found to metabolize readily through phase II and phase III pathways in *A. thaliana* cells. The rapid metabolism of DM-naproxen in plants may have contributed to its absence in the shoots.

Higher concentrations were consistently detected for M-acetaminophen than acetaminophen in both wheat roots and shoots (Fig. 3a). In wheat shoots, only M-acetaminophen was detected, suggesting that methylation rendered acetaminophen more mobile and a greater potential to translocate from roots to shoots. In general, DM-methylparaben was found to be taken up more rapidly than methylparaben into wheat roots and reached 20.66 ± 2.78 μg/g (d.w.) at 6 h after the treatment (Fig. 3c). In comparison, the highest level of methylparaben in roots was observed at 12.34 ± 1.33 μg/g after 96 h of exposure. However, methylparaben consistently exhibited much higher concentrations than DM-methylparaben in the shoots, suggesting a greater potential for translocation for methylparaben (Fig. 3c). Both compounds were found to undergo rapid metabolism, and their levels after 10 d of incubation were considerably lower than at earlier time points in the roots, while essentially no DM-methylparaben was found in the shoots. As the demethylated derivative of naproxen, although DM-naproxen was taken up quickly and reached 33.32 ± 8.41 μg/g in wheat roots after 24 h, it appeared to be rapidly metabolized (Fig. 3d), as only 0.33 ± 0.02 μg/g DM-naproxen was detected in the roots after 10 d. In comparison, naproxen was accumulated in both roots and shoots at consistently higher concentrations than DM-naproxen throughout the experiment (Fig. 3d). In wheat shoots, DM-naproxen was consistently below the detection limit, suggesting limited translocation, and/or rapid transformations in the roots via pathways such as conjugation.

Both DM-diazepam and diazepam showed significant accumulation in wheat plant (Fig. 3b). At the end of 10-d exposure, 32.74 ± 0.64 μg/g and 13.12 ± 2.79 μg/g of diazepam were detected in roots and shoots, respectively, while the corresponding values were 15.36 ± 1.51 μg/g and 11.81 ± 0.40 μg/g for DM-diazepam, suggesting active translocation after entry in the roots. Among the four pairs of compounds considered in this study, diazepam and DM-diazepam have the largest log *K*_ow_ (Table 1). Between diazepam and DM-diazepam, the root accumulation of DM-diazepam was greater than diazepam during the first few sampling time points; however, an opposite trend was observed after 48 h of incubation, where the level of diazepam appeared to be significantly greater than DM-diazepam (Fig. 3b). Levels of both diazepam and DM-diazepam in the shoots increased over time, and there was no statistically significant difference between their concentrations at the same time points. It must be noted that unlike the other compounds considered in this study, demethylation of diazepam does not introduce a hydroxyl group into the structure. Therefore the almost identical accumulation of diazepam and DM-diazepam may be attributed to their similar physicochemical properties (Table 1).

As CECs and their methylated or demethylated derivatives were taken up by wheat seedlings, their levels in the nutrient solution decreased (Fig. S2). The rate of dissipation was similar between acetaminophen and M-acetaminophen, and between methylparaben and DM-methylparaben. However, diazepam and naproxen appeared to decline at a slower rate than their demethylated counterparts (Fig. S2). Consequently, the estimated *T*_1/2_ values were also significantly longer for diazepam and naproxen than their demethylated derivatives (Table 2). The prolonged availability of diazepam and naproxen in the nutrient solution may have contributed to their relatively high accumulation in wheat seedlings (Fig. 3b and 3d).

Translocation factor (TF) in the whole wheat plant was calculated as the ratio of the concentration in shoots to that in roots (C_shoot_/C_root_) at the end of exposure (Briggs et al., 1982; Keerthanan et al., 2021; Trapp, 2000; Wu et al., 2013):

(1) $$\text{Translocation Factor }(TF)=\frac{\text{Concentration in shoots}}{\text{Concentration in roots}}$$

**Fig. 4.**
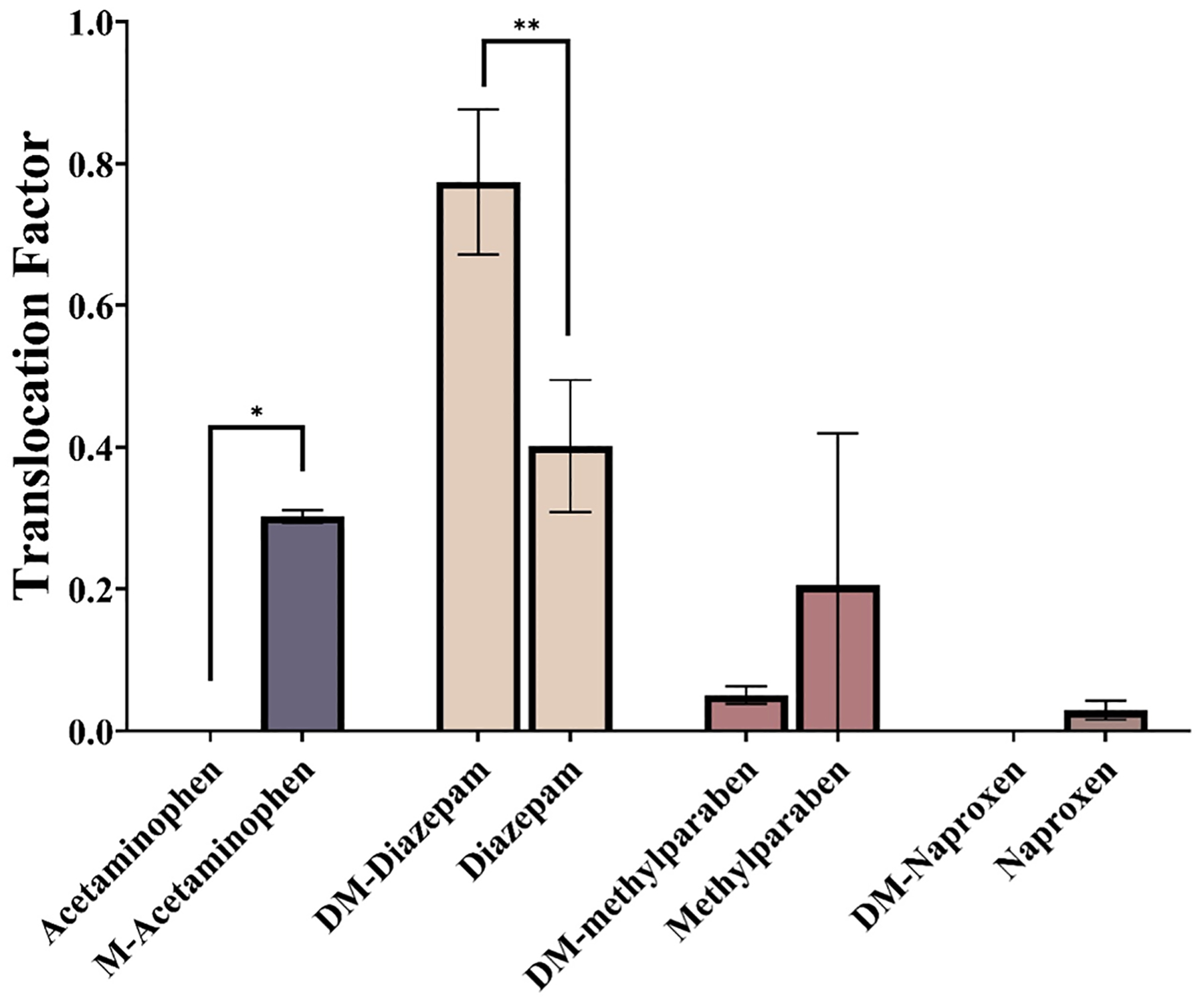
Translocation factor (TF) of test compounds and their methylated or demethylated counterparts in wheat seedlings at 10 d.

The derived TF values of the four pairs of compounds are shown in Fig. 4. The TF values for all target compounds in this study were less than 1, reflecting generally limited mobility from roots to shoots. With the exception of diazepam and DM-diazepam, TF values were generally greater for the methylated compound than the demethylated counterpart, although the difference was statistically significant only between acetaminophen and M-acetaminophen. For example, the derived TF was 0.30 ± 0.01 for M-acetaminophen, while the TF for acetaminophen was 0 as acetaminophen was not found in the shoots (Fig. 3a). However, diazepam and DM-diazepam showed an opposite trend, where DM-diazepam exhibited a greater TF (0.77 ± 0.10) than its methylated counterpart diazepam (0.40 ± 0.09), and the difference was statistically significant (*p* < 0.01, Fig. 4). As noted above, demethylation of diazepam does not lead to significant changes in physicochemical properties, and in fact, log *K*_ow_ 2.93 of DM-diazepam was slightly greater than that for diazepam (log *K*_ow_ 2.82) (Table 1). These results suggest that the effect of methylation or demethylation on the translocation of CECs in whole plants is specific to the molecular structure of individual compounds, and the changes that the reaction brings to the compound’s properties, such as hydrophobicity. When demethylation results in increased polarity (or decreased hydrophobicity), reduced plant uptake and translocation may be expected. In contrast, methylation generally leads to increased hydrophobicity and may be expected to contribute to enhanced plant uptake and translocation.

### 3.3. Correlation between accumulation and physicochemical properties

Physicochemical properties of organic compounds, such as hydrophobicity (indicated by *K*_ow_, partition coefficient between octanol and water) and ionization, are known to greatly influence their accumulation in organisms (Gao et al., 2022; Li et al., 2022; Shahriar et al., 2021). Several previous studies reported a positive linear relationship between log *K*_ow_ and root accumulation in various plant species for neutral xenobiotics (Briggs et al., 1982; Li et al., 2022; Miller et al., 2016; Wu et al., 2013). For ionic xenobiotics, the situation is more complicated. On the one hand, charged compounds are generally less accumulative than neutral species, especially anions, because the cell membranes are negatively charged. On the other hand, ionic species may interact with cell walls and membranes, involving in processes such as “ion trap”, which may contribute to more accumulation in plants (Miller et al., 2016; Shi et al., 2022; Trapp, 2009). For ionizable xenobiotics, it is important to determine their fraction of neutral species (*f*_*n*_) in order to predict their bioaccumulation potential in plants, since neutral molecules are usually considered to be taken up more readily by plants than their ionized forms (Trapp, 2000; Wu et al., 2013). Therefore, p*K*_a_ and the ambient pH are important factors regulating the plant uptake of ionizable compounds. In this study, pH of the plant cell culture solution and the whole wheat hydroponic culture solution were measured to be 5.80 and 5.10, respectively. The fraction of the neutral species (*f*_*n*_) for the compounds in the culture media was calculated using (Trapp, 2009; Wu et al., 2013):

(2) $$f_{n}=\frac{1}{1+10^{i(pH-pK_{a})}}$$

where *i* is 1 for acids and −1 for bases.

By considering *K*_ow_ for the neutral species and the dissociation rate of ionizable compounds, the pH-adjusted octanol–water partition coefficient log *D*_ow_ was estimated as (Table 1):

(3) $$log\;D_{\text{ow}}=log\;K_{\text{ow}}+log\;f_{n}$$

As log *D*_ow_ discounts for the ionized fraction, it is expected to correlate more closely with bioaccumulation than log *K*_ow_ for ionizable compounds.

Among the four pairs of compounds considered here, methylation and demethylation had varied effects on the accumulation in *A. thaliana* cells for the different CECs, and the effect was molecule-specific. In wheat seedlings, the demethylated derivative in each pair, when demethylation causes the introduction of a polar functional group (e.g., hydroxyl group), was accumulated at a comparatively reduced level and also exhibited more limited translocation. The limited accumulation of demethylated derivatives, especially in the shoots, may be partly attributable to their rapid subsequent metabolism, such as conjugation. Demethylation of M-acetaminophen, methylparaben and naproxen led to the introduction of a hydroxyl or carboxyl group into the molecule, resulting in lower log *K*_ow_ and log *D*_ow_ values than those for their parent form (Table 1). As demonstrated in previous studies, CECs with functional groups such as hydroxyl group are highly susceptible to conjugation with biomolecules in plants, including amino acids, sugars, and sulfate (Fu et al., 2017b, 2017a; LeFevre et al., 2015; MacHerius et al., 2012). In contrast, the *N*-demethylated diazepam derivative, DM-diazepam, has a slightly greater log *K*_ow_ (2.93) than diazepam (2.82) (Table 1). The high similarity in log *K*_ow_ and log *D*_ow_ between diazepam and DM-diazepam may explain their almost identical accumulation in *A. thaliana* as well as in wheat seedlings. Therefore, when demethylation leads to a decreased log *K*_ow_ or log *D*_ow_ value for the compound (as observed for *O*-demethylation in this study), it may generally result in reduced plant accumulation. The reduced accumulation may be caused by a decrease in uptake into the root because of the increased polarity, and/or rapid Phase II transformations such as conjugation with endogenous biomolecules in plants. Conversely, when a compound becomes methylated and its log *K*_ow_ or log *D*_ow_ increased, as in the case of conversion of acetaminophen to M-acetaminophen (i.e., p-acetanisidine), DM-naproxen to naproxen, and DM-methylparaben to methylparaben, plant uptake and translocation are likely enhanced.

**Fig. 5.**
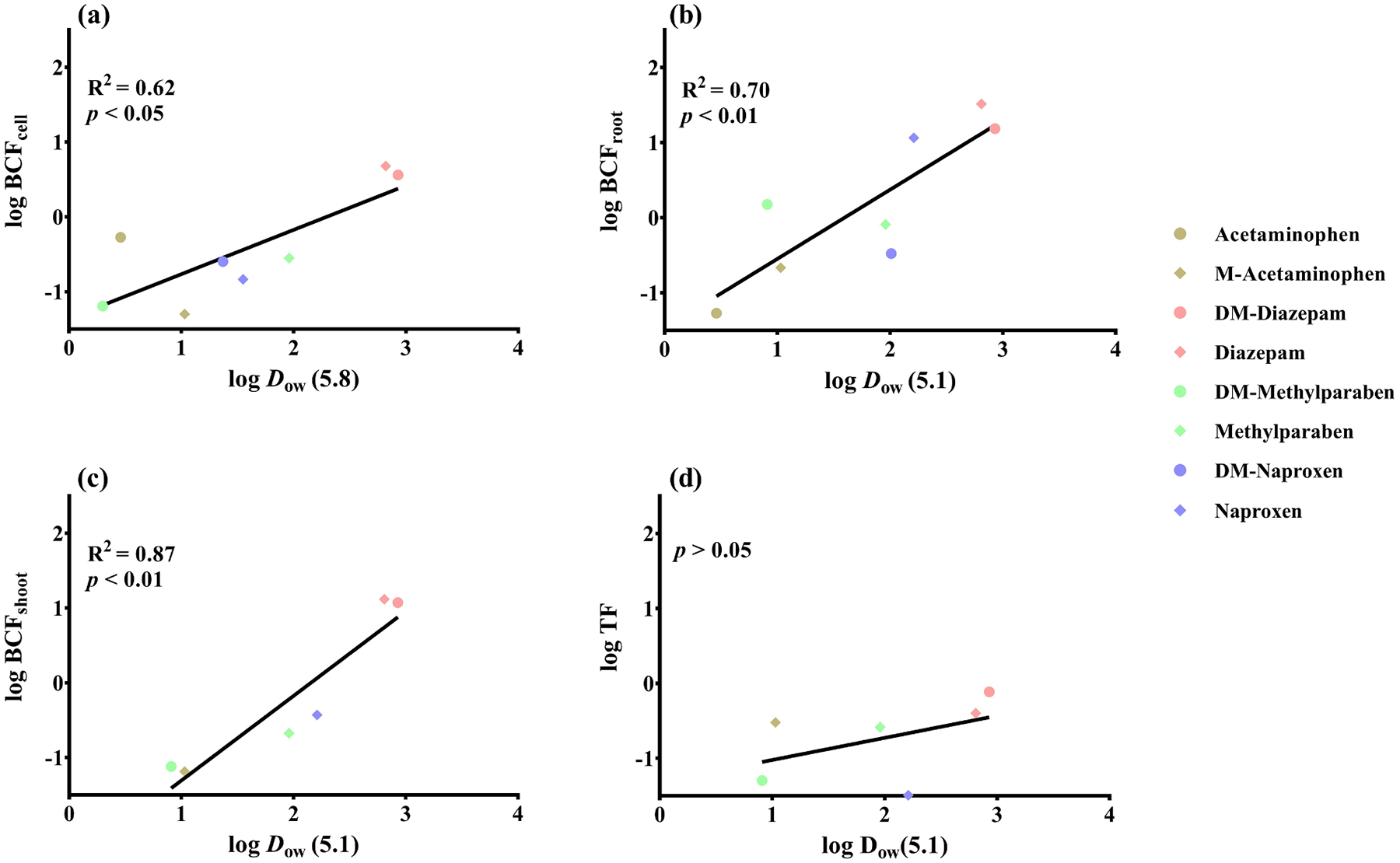
Correlations between log *D*_ow_ and (a) bioconcentration factor in *A. thaliana* cells, (b) bioconcentration factor in wheat root, (c) bioconcentration factor in wheat shoot, and (d) translocation factor in wheat seedlings.

To better understand the relationships between physicochemical properties of CECs and their accumulation in plants, the bioconcentration factor (BCF), calculated as the ratio of chemical concentration in *A. thaliana* cells, wheat roots or shoots at the end of exposure, to the initially spiked concentration, and log TF in wheat seedlings for all target compounds are plotted against their log *D*_ow_ values (Fig. 5). Positive linear relationships were observed between log BCF and log *D*_ow_ for the different treatments in this study (Fig. 5a, b, and c, *p* < 0.05), indicating that the bioaccumulation of methylated or demethylated CEC derivatives in plants was closely related to the pH adjusted hydrophobicity parameter log *D*_ow_. This linear relationship between log *D*_ow_ and accumulation was also observed for vegetables grown hydroponically in previous studies (Hyland et al., 2015; Miller et al., 2016; Wu et al., 2013). Therefore, differences in accumulation by *A. thaliana* cells or wheat seedlings caused by methylation or demethylation may be largely explained by the change imparted on *K*_ow_ or *D*_ow_.

No significant correlation was found between log TF and log *D*_ow_ in this study (Fig. 5d, *p* > 0.05), likely due to the limited number of compounds considered in this study. Wu et al. (2013) observed a generally negative correlation for pharmaceuticals and personal care products in lettuce, spinach, cucumber and pepper. Another study conducted by Li et al. (2018) did not show any significant correlation between log TF and log *K*_*ow*_ for neonicotinoids in Japanese mustard. Different treatments, plant species and compounds were used in those studies, suggesting that the translocation of xenobiotics in plants may be affected by not only the physicochemical properties of the xenobiotics, but also the inherent characteristics of plants. In addition, plants have a cascade of enzymes that are capable of facilitating metabolic transformations, and metabolism affects TF, as rapid metabolism in the root would translate into a diminished TF. Also, weak acidic CECs dissociated in the cytosol could be repelled by the negatively charged cell membranes, and therefore, become “trapped” in root cells, which may also limit their translocation (Keerthanan et al., 2021). Active metabolism, such as conjugation with endogenous plant biomolecules, and the possible “ion trap” in root cells, likely contributed to the lack of apparent translocation for acetaminophen, DM-naproxen and DM-methylparaben in this study.

While physicochemical parameters such as p*K*_a_ and *K*_ow_ are available for many man-made compounds, they are often unknown for TPs. In the absence of experimentally measured values, models based on molecular descriptors may be used for obtaining approximate physicochemical properties for TPs. For example, ChemAxon provides a calculator for predicting log *K*_ow_, p*K*_a_, and log *D*_ow_ of organic compounds. In the case of bisphenol A (BPA), methylation may be predicted to increase its log *D*_ow_ (pH at 5) from 4.04 to 4.19 for BPA monomethyl ether and further to 4.34 for BPA dimethyl ether. Likewise, while TBBPA has a log *D*_ow_ (pH at 5) of 7.11, it increases to 7.41 for TBBPA dimethyl ether. Methylation of diclofenac to diclofenac-methyl ether increases its log *D*_ow_ (pH at 5) from 3.21 to 4.4; log *D*_ow_ of methyl triclosan (5.13) is also greater than triclosan (4.98). However, methylation of compounds does not always result in increased log *D*_ow_ values. For example, caffeine (−0.55) would show a smaller log *D*_ow_ than some of its demethylated products, i.e., paraxanthine (0.24) and 7-methylxanthine (0.02). Hence, methylation and demethylation change the physicochemical properties of CECs, and the change induced is highly molecule-specific. Tools like ChemAxon could help predict basic properties of organic compounds, including TPs that do not always have experimentally derived values. It is feasible to incorporate changes in physicochemical properties, using either experimentally derived or estimated values, into well-established empirical relationships to evaluate the potential influence of common transformation reactions such as methylation and demethylation on plant uptake for a large range of CECs in the scenarios of beneficial reuse of treated wastewater effluent and biosolids.

## 4. Environmental implications

Simple reactions such as methylation and demethylation are common abiotic and biotic transformations which contribute to the co-occurrence of many TPs of man-made chemicals in the environment. As demonstrated in this study, methylation and demethylation could result in changes in a chemical’s physicochemical properties, and the magnitude of change is specific to the molecule and the types of functional groups undergoing the conversion. The changes in a chemical’s physicochemical properties could subsequently lead to different environmental behaviors and risks, such as accumulation and translocation in higher plants. Moreover, a methylated or demethylated derivative may have increased or decreased biological activity as compared to the parent compound. Given that there are numerous CECs in sources such as treated wastewater and biosolids, the co-existence of additional TPs presents another layer of challenge to the risk assessment of man-made chemicals. Although not explored in this study, differences may be similarly expected in microbial degradation and hence persistence of TPs, as well as bioaccumulation and toxicity to non-target organisms, such as aquatic and terrestrial invertebrates. For instance, methylated diclofenac showed a 430-fold increase in acute toxicity to *Hyalella azteca* compared to diclofenac (Fu et al., 2020). BPA mono- and dimethyl ethers were also found to result in enhanced mortality and developmental toxicity in zebrafish embryos than BPA (McCormick et al., 2011). Methyl triclosan was shown to exhibit greater bioaccumulation in snails but reduced bioaccumulation in algae compared to triclosan (Coogan et al., 2007; Coogan and la Point, 2008). The potential influence of methylation and demethylation on the phytotoxicity of CECs was not explored in this study; further research should consider this aspect by evaluating changes in enzyme activities and photosynthetic efficiency, among other endpoints (Sun et al., 2019).

It must be noted that the experiments in this study were conducted under simplistic conditions. More processes and variables are involved in the soil–plant system under field conditions and their interactions likely determine the ultimate fate and risks of a chemical. For instance, methylation or demethylation may alter a compound’s stability in the rhizosphere as well as its adsorption to the soil solid phase, which in turn influence the chemical’s availability for plant uptake. As a chemical’s log *K*_ow_ increases, its adsorption to soil increases while its presence in the soil porewater decreases, leading to a reduced availability for uptake into plant roots. The interactions of these fate and transport processes in the soil–plant system may therefore amplify or diminish the effects brought upon by the transformations and should be further studied under field-relevant conditions.

A significant bottleneck to the holistic assessment of environmental risks is the sheer number of CECs and the fact that experimentally determined physicochemical properties are often not available for their transformation intermediates. It is likely that for many CECs, transformation reactions consistently lead to reduced biological availability and lower non-target toxicity. In this case, only certain transformation reactions for a subset of CECs may pose an increased risk. Predicting essential physicochemical parameters such as p*K*_a_ and log *D*_*ow*_ using well-established chemical calculation tools may generate the first line of information for identifying TPs with enhanced potential for bioaccumulation or non-target toxicity. This approach may be used to effectively direct future research efforts to better understand the environmental significance of common transformation reactions for CECs.

## Supplementary Material

Supplementary Materials

## Figures and Tables

**Table 1:** Physicochemical properties of selected CECs and their methylated or demethylated counterparts.

			pH 5.8		pH 5.1	
Compound	log *K*_ow_^a^	p*K*_a_^c^	*f*_n_	log *D*_ow_^d^	*f*_n_	log *D*_ow_^d^
Acetaminophen	0.46	9.38	1.00	0.46	1.00	0.46
M-Acetaminophen	1.03	1.5^b^	1.00	1.03	1.00	1.03
DM-Diazepam	2.93	2.85^b^	1.00	2.93	0.99	2.93
Diazepam	2.82	3.40	1.00	2.82	0.98	2.81
DM-Methylparaben	1.58	4.54	0.05	0.30	0.22	0.91
Methylparaben	1.96	8.34	1.00	1.96	1.00	1.96
DM-Naproxen	2.84^b^	4.34^b^	0.03	1.37	0.15	2.01
Naproxen	3.18	4.18	0.02	1.55	0.11	2.21

a Measured values collected from PubChem: https://pubchem.ncbi.nlm.nih.gov/.

b Predicted by ChemAxon and collected from The Human Metabolome Database: https://hmdb.ca/.

c Measured value from CompTox Chemicals Dashboard: https://comptox.epa.gov/dashboard/.

d Calculated log*D*_ow_ values crosschecked with the log*D*_ow_ values predicted by ChemAxon: https://disco.chemaxon.com/calculators/demo/plugins/logd/.

**Table 2:** The dissipation half-life of test compounds in *A. thaliana* cell culture media and wheat seedling hydroponic solution.

Compound	Dissipation half-life (T_1/2_, h)	
	*A. thaliana* cell media	Hydroponic solution
Acetaminophen	20.4 (14.0–30.3)^a^	11.6 (7.2–19.7)
M-Acetaminophen	34.0 (23.69–50.0)	13.7 (6.8–30.2)
DM-Diazepam	49.7 (23.1–146.0)	36.2 (9.0–109.9)
Diazepam	106.2 (46.2–988.1)	245.5 (194.5–323.8)
DM-Methylparaben	0.69 (0.68–0.70)	8.2 (5.3–13.2)
Methylparaben	1.05 (0.42–1.58)	12.2 (6.3–26.9)
DM-Naproxen	N.A.^b^	8.3 (6.0–11.7)
Naproxen	0.9 (0.7–1.0)	67.4 (25.1–174.7)

a Values expressed as “the best fit value (95 % CI)”.

b N.A. - not available due to extremely rapid dissipation.

## Data Availability

All the data were included in the figures and tables in the manuscript and supporting information.
