# Supplementary material for: Influence of methylation and demethylation on plant uptake of emerging contaminants
Source: Environ Int. Author manuscript; Available in PMC 2023 Dec 1. (PMC9988749; doi:10.1016/j.envint.2022.107612)
Supplement: Supplementary Materials [file NIHMS1878708-supplement-Supplementary_Materials.docx]

Supplementary Materials

Influence of Methylation and Demethylation on Plant Uptake of Emerging Contaminants

Yaxin Xiong, Qingyang Shi, Nathan Darlucio Sy, Nicole M. Dennis, Daniel Schlenk, Jay Gan^*^

**Author Affiliations**

Department of Environmental Sciences, University of California, Riverside, CA 92521, USA.

***Corresponding author**: Dr. Jay Gan

Department of Environmental Sciences, University of California, Riverside, CA 92521, USA.

Tel: (951) 827-2712; E-mail: [jgan@ucr.edu](mailto:jgan@ucr.edu)

# Text S1. NT-1 media

The *A. thaliana* suspension cell culture solution was prepared with the following ingredients: 4.3 g Murashige and Skoog basal salt mixture (MS), 30 g sucrose, 0.18 g KH_2_PO_4_, 100 mg myo-Inositol, 220 µL of 2 mg/mL 2,4-D stock solution and 100 µL of 10 mg/mL Thiamine stock solution. These components were dissolved in 800 mL deionized water. Then the pH of the solution was adjusted to 5.8 with 5 M NaOH solution. Finally, the volume of the solution was adjusted to 1 L. An aliquot (75 mL) of the final solution was added to a 250 mL glass flask, and autoclaved for 20 mins. After it was cooled down to room temperature, 15 mL of *A. thaliana* suspended cells was added into flasks, and then maintained at 24 °C and 130 rpm with constant light for 7 days before it can be sub-cultured.

#

# Table S1. MRM transitions of target compounds on UPLC-MS/MS

| Compound | MRM (m/z) | | | |
| --- | --- | --- | --- | --- |
|  | Quantification | CV/CE* | Qualification | CV/CE |
| ESI+ |  |  |  |  |
| Acetaminophen | 151.97 > 109.99 | 38/22 |  |  |
| M-Acetaminophen | 166.03 > 124.07 | 38/22 | 166.03 > 92.74 | 38/24 |
| *d4*-Acetaminophen | 156.03 > 113.99 | 40/12 | 156.03 > 96.75 | 40/22 |
| DM-diazepam | 271.03 > 139.99 | 56/28 | 271.03 > 165.03 | 56/28 |
| Diazepam | 285.03 > 154.02 | 56/26 | 285.03 > 193.09 | 56/32 |
| *d5*-Diazepam | 290.10 > 198.07 | 54/34 | 290. 10 > 154.11 | 54/26 |
| ESI- |  |  |  |  |
| DM-Methylparaben | 137.09 > 93.08 | 34/15 |  |  |
| Methylparaben | 151.05 > 92.03 | 38/20 | 151.05 > 136.00 | 38/14 |
| *d4*-Methylparaben | 155.05 > 96.05 | 36/20 | 155.05 > 140.01 | 36/14 |
| DM-Naproxen | 215.15 > 171.15 | 21/6 | 215.15 > 169.15 | 21/28 |
| Naproxen | 229.15 > 185.15 | 17/8 | 229.15 > 170.15 | 17/16 |
| *d3*-Naproxen | 232.18 > 188.10 | 14/5 | 232.18 > 173.14 | 14/18 |

*CV-cone voltage (kV), CE-collision energy (eV).

# Table S2. Detection limits and recoveries of target compounds

| Compound | LOQ* ng/mL | Recovery (%) | | | | |
| --- | --- | --- | --- | --- | --- | --- |
|  |  | *A. thaliana* cells | Wheat roots | Wheat shoots | Cell culture media | Wheat hydroponic solution |
| Acetaminophen | 0.5 | 93.3 ± 7.7 | 83.2 ± 1.0 | 78.1 ± 0.7 | 112.0 ± 6.1 | 113.4 ± 5.7 |
| M-Acetaminophen | 0.2 | 63.4 ± 7.1 | 63.8 ± 13.3 | 63.2 ± 1.1 | 96.0 ± 2.7 | 98.8 ± 4.0 |
| DM-Diazepam | 0.2 | 82.5 ± 1.7 | 70.7 ± 3.8 | 60.0 ± 3.7 | 75.1 ± 2.3 | 77.3 ± 1.9 |
| Diazepam | 0.25 | 95.9 ± 8.9 | 83.3 ± 10.2 | 69.5 ± 11.5 | 114.9 ± 6.7 | 85.0 ± 13.0 |
| DM-Methylparaben | 3.0 | 80.8 ± 6.7 | 42.4 ± 10.1 | 54.8 ± 4.7 | 101.0 ± 5.8 | 100.8 ± 1.5 |
| Methylparaben | 1.5 | 64.3 ± 6.9 | 100.7 ± 2.8 | 97.4 ± 1.7 | 126.2 ± 3.2 | 97.3 ± 0.7 |
| DM-Naproxen | 3.0 | 65.0 ± 5.2 | 39.9 ± 7.3 | 42.6 ± 6.3 | 90.3 ± 2.5 | 90.3 ± 9.6 |
| Naproxen | 2.0 | 115.8 ± 3.0 | 89.1 ± 2.8 | 84.5 ± 3.6 | 80.3 ± 6.6 | 100.5 ± 1.3 |

*LOQ, limit of quantification.


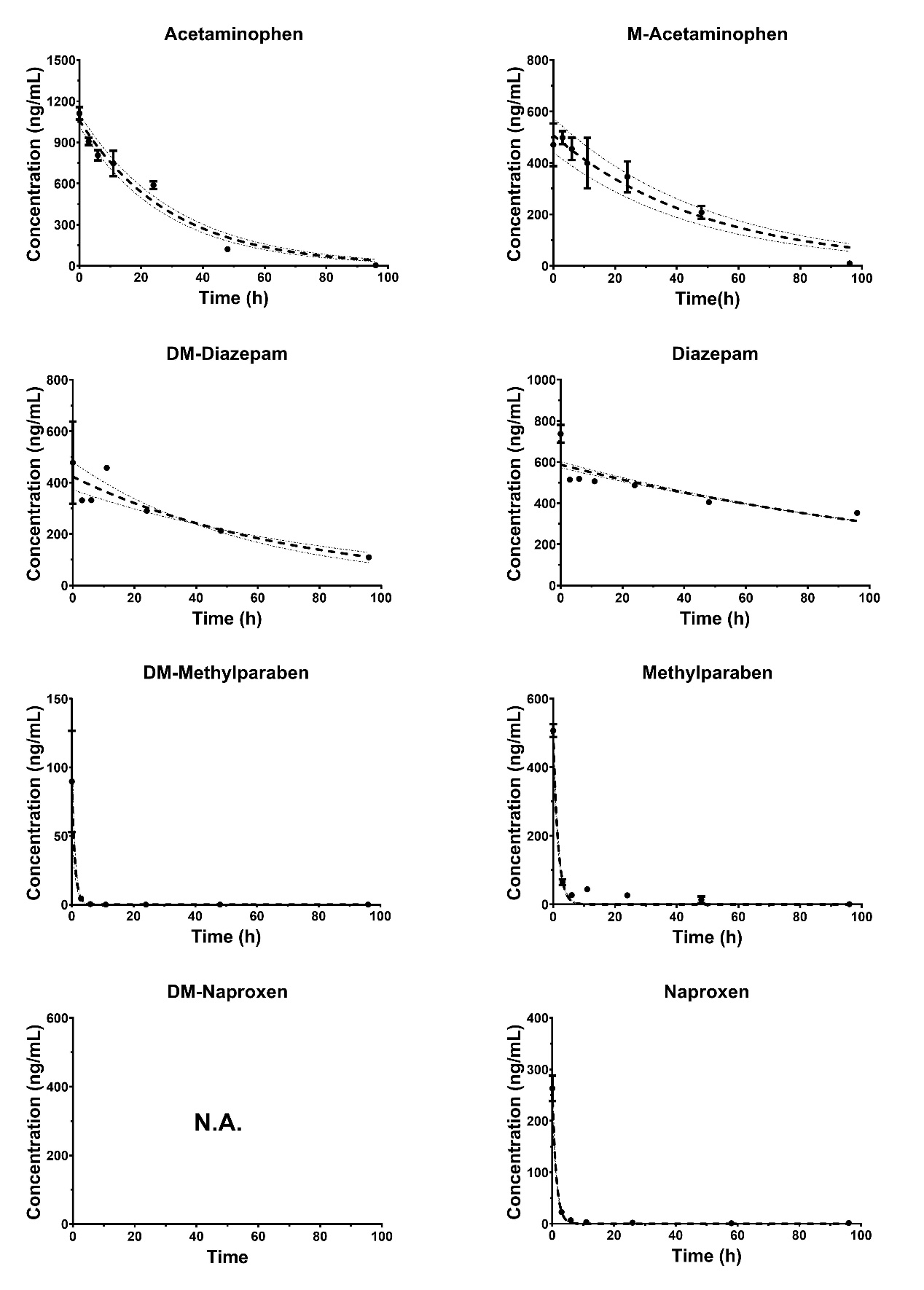


# Figure S1. The first-order dissipation kinetics of target compounds in plant cell culture media.


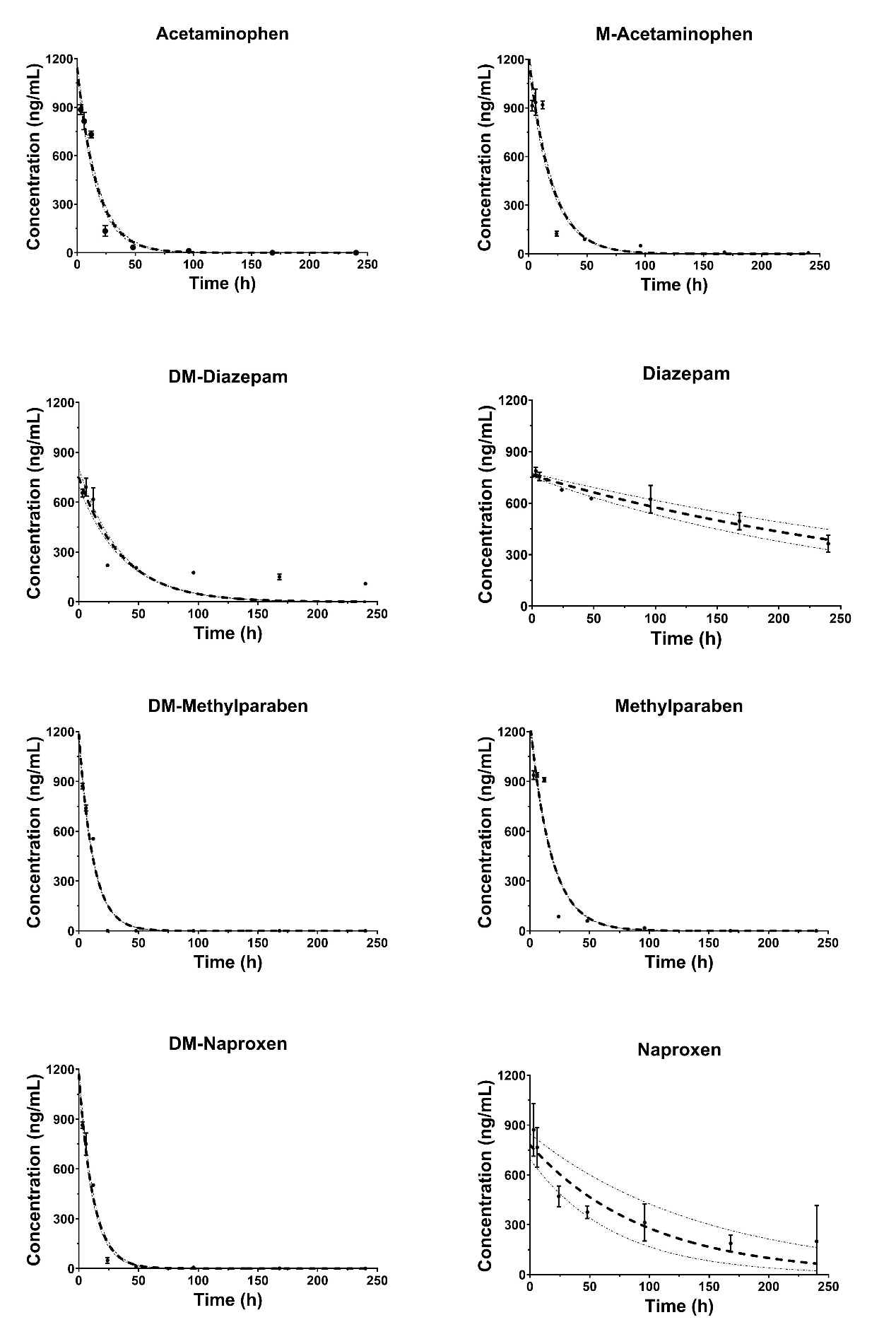


# Figure S2. The first-order dissipation kinetics of target compounds in wheat hydroponic solutions.
